# Pre-Procedural Atorvastatin Mobilizes Endothelial Progenitor Cells: Clues to the Salutary Effects of Statins on Healing of Stented Human Arteries

**DOI:** 10.1371/journal.pone.0016413

**Published:** 2011-01-25

**Authors:** Benjamin Hibbert, Xiaoli Ma, Ali Pourdjabbar, Trevor Simard, Katey Rayner, Jiangfeng Sun, Yong-Xiang Chen, Lionel Filion, Edward R. O'Brien

**Affiliations:** 1 University of Ottawa Heart Institute, Ottawa, Ontario, Canada; 2 Department of Biochemistry Microbiology and Immunology, Faculty of Medicine, University of Ottawa, Ottawa, Ontario, Canada; Universität Würzburg, Germany

## Abstract

**Objectives:**

Recent clinical trials suggest an LDL-independent superiority of intensive statin therapy in reducing target vessel revascularization and peri-procedural myocardial infarctions in patients who undergo percutaneous coronary interventions (PCI). While animal studies demonstrate that statins mobilize endothelial progenitor cells (EPCs) which can enhance arterial repair and attenuate neointimal formation, the precise explanation for the clinical PCI benefits of high dose statin therapy remain elusive. Thus we serially assessed patients undergoing PCI to test the hypothesis that high dose Atorvastatin therapy initiated prior to PCI mobilizes EPCs that may be capable of enhancing arterial repair.

**Methods and Results:**

Statin naïve male patients undergoing angiography for stent placement were randomized to standard therapy without Atorvastatin (n = 10) or treatment with Atorvastatin 80 mg (n = 10) beginning three days prior to stent implantation. EPCs were defined by flow cytometry (e.g., surface marker profile of CD45dim/34+/133+/117+). As well, we also enumerated cultured angiogenic cells (CACs) by standard *in vitro* culture assay. While EPC levels did not fluctuate over time for the patients free of Atorvastatin, there was a 3.5-fold increase in EPC levels with high dose Atorvastatin beginning within 3 days of the first dose (and immediately pre-PCI) which persisted at 4 and 24 hours post-PCI (p<0.05). There was a similar rise in CAC levels as assessed by *in vitro* culture. CACs cultured in the presence of Atorvastatin failed to show augmented survival or VEGF secretion but displayed a 2-fold increase in adhesion to stent struts (p<0.05).

**Conclusions:**

High dose Atorvastatin therapy pre-PCI improves EPC number and CAC number and function in humans which may in part explain the benefit in clinical outcomes seen in patients undergoing coronary interventions.

## Introduction

Percutaneous coronary intervention (PCI) is the preferred revascularization strategy for patients with coronary artery disease. Recent developments with drug eluting stents (DESs) have reduced the need for revascularization compared to balloon angioplasty and bare metal stents (BMSs) resulting in less need for repeat revascularization. Current strategies for reducing in-stent neointima formation commonly exploit the anti-proliferative and anti-inflammatory effects of paclitaxel and sirolimus. Unfortunately, the negative effect of these drugs on stent strut endothelialization may be dire due to an increased risk of sudden stent thrombosis.[1] Clinical and animal studies suggest a role for circulating endothelial progenitor cells (EPCs) in reconstituting the endothelium and reducing neointima formation following injury. [2] Indeed, the paucity and/or impaired functional capacity of EPCs are inversely associated with cardiac risk factors, cardiovascular outcomes and restenosis rates.[3], [4] In animal studies, interventions that enhance mobilization of EPCs such as G-CSF, HMG-CoA reductase inhibitors (also known as “statins”) or estrogens uniformly result in improved endothelialization and diminished neointimal formation.[5]–[7]

Statins induce a robust mobilization of murine CD117+/Sca-1+ progenitor cells and EPCs via the PI3K/Akt pathways and enhance endothelialization of injured vessels thereby leading to attenuation of neointimal formation.[7]–[10] Moreover, recent clinical trials show an LDL-independent superiority of intensive compared to moderate statin therapy in reducing target vessel revascularization in patients who undergo PCI for acute coronary syndromes.[11] However, the precise explanation for the clinical advantage of high dose statin therapy with PCI remains elusive. Therefore, we performed serial assessments of patients undergoing PCI to test the hypothesis that high dose Atorvastatin (80 mg) therapy initiated prior to PCI mobilizes EPCs that may be capable of enhancing arterial repair.

## Methods

### Ethics Statement

The protocol was approved by the University of Ottawa Heart Institute Research Ethics Committee (protocol #:04-122) and Health Canada. All patients gave written informed consent and research was conducted according to the principles expressed in the Declaration of Helsinki.

### Patients and Protocol

Our study was designed to longitudinally assess the effects of statin therapy on patients undergoing PCI with stent deployment. Patient inclusion criteria included the following: >18 years of age, no treatment with a HMG-Co-A reducatse inhibitor in the preceding 3 months, availability for a 14 day follow-up blood test, and the patient had to be scheduled for a coronary angiogram with possible *ad hoc* PCI and stent implantation. Furthermore, to exclude the confounding factor of estrogens in pre- and post-menopausal women, only males were included in the study. Exclusion criteria included an unstable condition requiring urgent cardiac catheterization, hematological malignancy, therapy with G-CSF or GM-CSF, or previous statin intolerance.

Patients were randomized to Atorvastatin 80 mg per day or medical therapy without a statin beginning three days prior to a scheduled angiogram. Baseline laboratory tests included a complete blood count, HbA1C level, fasting lipid profile, C-reactive protein (CRP), erythrocyte sedimentation rate (ESR), assessment of EPCs by flow cytometry (CD45dim/CD34+/CD133+/CD117+), and enumeration of cultured angiogenic cells (CACs). All of the aforementioned tests were repeated at time of arterial catheterization (i.e., at outset of coronary angiographic procedure and prior to commencement of an *ad hoc* PCI), 4 hours post PCI, 24 hours post PCI, and at 14-day follow-up. In addition, troponin-T (TnT) and creatine kinase-MB (CKMB) fractions were measured pre-procedural and at the 4 and 24 hour follow-up time points to identify peri-procedural myocardial infarctions (MI) as defined by a>3x rise above the upper limit of normal.

Statin naïve patients were randomized in blocks of 6; however, after the first 18 patients had been enrolled 8 patients had completed the protocol in the non-statin control arm and only 2 patients had completed the protocol in the statin arm. In the statin arm of the study the following patients did not complete the protocol: 2 patients without CAD, 3 patients who were deemed non-revascularizable and 2 patients who were referred for coronary artery bypass grafting. Therefore, the statin randomization scheme was redone for a total of 40 patients to be enrolled in order to yield 10 patients completing each study arm. As well, 11 healthy controls without clinically evidence of CAD were recruited for comparison of baseline EPC and CAC levels. Four patients initially randomized in the trial whom were found to have normal coronaries were combined with the 11 healthy individuals for a total of 15 healthy controls.

### EPC and CAC quantification

EPCs were assessed by flow-cytometry and CACs by *in vitro* culture assay. All blood samples for EPC enumeration were drawn by venous or arterial puncture then anticoagulated with EDTA.

EPCs were enumerated using a standardized flow cytometric protocol. Briefly, five-color flow cytometry was performed for the markers CD34 (clone 581, fluorochrome PC7), CD45 (clone J133, fluorochrome ECD), CD133 (clone AS133, fluorochrome PE), and CD117 (clone 104D2D1, fluorochrome PC5), and CD49e (clone SAM1, fluorochrome FITC). Antibodies were purchased from Beckman Coulter and studies performed on a Beckman Coulter Cytomics FC 500 cytometer. Red blood cell lysis was performed using IO Test 3 solution (Beckman Coulter) and samples were then incubated with appropriate dilutions of antibodies. As isotype controls are known to mask rare cell populations,[12] none were used in this analysis, and baseline fluorescence was determined using unstained cells. All reported EPC counts were adjusted for total white blood cell counts as determined by standard complete blood counts in our regional hematology laboratory.

We prospectively defined EPCs as CD45_dim_/CD34+/CD133+/CD117+. Recognizing that circulating EPCs have historically been defined by CD34+/KDR+[13], [14] preliminary studies were performed looking at expression of KDR and CD117 cells which demonstrated that virtually all cells expressing KDR also co-expressed CD117. Thus, KDR was not included in the final panel of markers. It is important to note that human CD45-/CD117+/KDR+ cells can incorporate into the coronary vasculature and display clonal proliferative potential, suggesting an EPC phenotype.[13], [15] Moreover, CD45-/CD117+ of cardiovascular origin have recently been shown to co-express CD31, CD34, CXCR4, and KDR.[13]

While initially termed EPCs, CACs are now recognized to be involved in regulating angiogenesis without directly contributing to post-natal vasculogenesis and thus are now termed cultured angiogenic cells. CAC have been extensively described in the literature and are known to be inversely associated with risk factors for CAD.[16]Indeed, numerous studies have highlighted the important roles CACs play in regulating the angiogenic and tissue remodeling response in various disease states.[17]–[19] The methodology used for the CAC culture assay is previously described.[20]–[23] Briefly, peripheral blood mononuclear cells (5×10^6^) isolated by Ficoll centrifugation were cultured in EGM-2 media (Cambrex) before being dispersed on fibronectin coated plates. Cells were washed at 4 days and adherent cells were maintained for 7 days prior to enumeration. For the task of enumeration, CACs were defined as cells dually positive for AcLDL uptake and *ulex europeus* agglutinin I (UEAI) binding. While uptake of AcLDL and binding of UEAI may non-specifically identify myeloid and epithelial cells,[13], [24] we have previously demonstrated the capacity of these cells to facilitate vascular repair *in vivo* [19] and others have demonstrated expression of the endothelial markers KDR, CD31, vWF, and eNOS.[13] DiI-AcLDL (2.5 µg/ml, Molecular Probes) was incubated with cultured CACs for 1 hour in a cell incubator. Subsequently, cells were washed and fixed with Cytofix Buffer (BD) and incubated with FITC-UEAI (5 µg/ml, Sigma) for 30 minutes. Plates of cells were again washed then incubated with a DAPI nuclear counterstain before a coverslip was applied to the well and double positive cells (CAC) were counted in 6 random high power fields (×200 magnification).

### CAC Functional assays

The effects of Atorvastatin on CAC survival, VEGF secretion, and adherence to bare metal stent struts were tested using 7 day old CACs. The culture media for Atorvastatin treated cells was supplemented with 0.1 µmol/L of Atorvastatin (Pfizer) or DMSO (vehicle) consistent with a previously published study.[25]

For cell survival, six high-power fields were enumerated and the media was changed every 4 days. Data are expressed as a percentage of initial cells. Secretion of VEGF was measured using a VEGF ELISA kit (R&D systems). CACs were plated in equal numbers and cultured in VEGF free EMG-2 media for 24 hours. Subsequently, 200 µL of the supernatant was isolated and used in the ELISA assay. Stent adhesion studies were performed using BMSs (Medtronic Micro-Driver) that were cut, flattened, and fixed to the bottom of 6 well plates using a Type-I collagen solution such that the stent strut projected above the collagen base as previously described.[22] Seven day old CACs (2×10^5^) were resuspended and cultured for 48 hours. Cells were stained with DAPI, fixed and attached cells enumerated.

Q-PCR for alpha integrins was performed as previously described.[19] Briefly, PCR was performed utilizing an annealing temperature of 56 degrees Celsius. The Qiagen QuantiTect SYBR PCR system was utilized as per manufacturer instructions. All experiments were performed using a Roche LightCycler 480. The following primer combinations were utilized: GAPDHfwd (CGCCTGGAGAAAGCTGCTAAGTAT), GAPDH rev (GCTTCACAAAGTGGTCATTGAGGG), α-1fwd (ACAAGTGACAGCGAAGAACCTCCT), α-1rev (TGGGTACAGCACAGGGTAACCATT), α-2fwd (ACTTTATCTCCAGCGGTACAAAGT), α-2rev (TGGGCCTTATCCCAATCTGACCAA), α-3fwd (CAAAGACAGGCAAACGGCAACGTA), α-3rev (TTATTGGTCGCGGTGAGAAGCCTA), α-4fwd (AGGGCAAGGAAGTTCCAGGTTACA), α-4rev (ACATGAGGACCAAGGTGGTAAGCA), α-4probe (AGCATTTATGCGGAAAGATGTGCGGG), α-5fwd (TGCCTGAGTCCTCCCAATTTCAGA), α-5rev (ACATGAGGACCAAGGTGGTAAGCA).

### Statistical Analysis

For statistical procedures, a p-value of <0.05 was considered significant. All continuous variables are expressed as means +/− standard error of the mean (SEM). Data normality was tested using the Kolmogorov-Smirnov test. Two way comparisons were done by *t* test and the interaction between statin therapy and PCI by two-way repeated measures analysis of variance with pairwise comparisons done with Bonferroni *post hoc* testing. Alternatively, for nonparametric statistical testing a Mann-Whitney Rank Sum test was used. All statistical procedures were performed using the Sigma Stat 3.5 statistical package.

## Results

### Clinical and laboratory profiles

In each group 10 patients completed the protocol receiving at least one stent. In total, 15 healthy controls were analyzed for comparison – 4 patients who underwent angiography and had normal coronary arteries and 11 healthy individuals without clinical evidence of CAD.

Of the 20 patients completing the protocol, all underwent successful revascularization with deployment of at least one stent. Baseline characteristics were similar between the treatment groups including the number and types of stents (DES vs BMS) used (**Table 1**). In terms of adverse events, only one post-PCI MI was recorded in the statin group and one patient in the control arm was withdrawn from the study after having a stroke related to the catheterization procedure. At time of discharge patients received evidence-based medical therapy for CAD (eg. aspirin, clopidogrel, β-blocker, and an angiotensin converting enzyme inhibitor or angiotensin II receptor blocker, **Table 1**). All patients that underwent PCI completed follow-up and no other adverse events were noted to 14 days.

**Table 1:** Patient Characteristics.

	Control Group	Statin Group	Healthy
	(n = 10)	(n = 10)	(n = 15)
*Characteristics*			
Males	10 (100%)	10 (100%)	15 (100%)
Age in years (SD)	59.5 (6.9)	63.0 (10.5)	34.4 (12.6)
CCS class I-II	5 (50%)	7 (70%)	0 (0%)
CCS class III-IV	5 (50%)	3 (30%)	0 (0%)
Previous PCI	2 (20%)	0 (0%)	0 (0%)
*Cardiac Risk Factors*			
Hypertension	3 (30%)	4 (40%)	0(0%)
Smoking	2 (20%)	2 (20%)	1 (6.7%)
Family History	3 (30%)	3 (30%)	2 (13.3%)
Hyperlipidemia	3 (30%)	3 (30%)	1 (6.7%)
Diabetes mellitus	2 (20%)	0 (0%)	0 (0%)
eGFR (SEM)	86 (7)	75 (6)	107 (9)
*Angiogram/PCI*			
# of stents deployed (SD)	1.2 (0.4)	1.6 (0.8)	
Drug-eluting stents used	4 (40%)	5 (50%)	
Severe CAD	0(0%)	2 (20%)	
*Medical Therapy*			
Clopidogrel	10 (100%)	10 (100%)	
Aspirin	10 (100%)	10 (100%)	
β-blocker	8 (80%)	9 (90%)	
ACEI/ARB	9 (90%)	10 (100%)	
Atorvastatin	0 (0%)	10 (100%)	

CCS Class – Canadian Cardiovascular Society Angina Class.

PCI – percutaneous coronary intervention.

CAD – coronary artery disease.

MI – myocardial infarction.

ACEI/ARB – angiotensin converting enzyme/angiotensin II receptor blocker.

eGFR – estimated glomerular filtration rate ml/min/1.73 m^2^.

SD – standard deviation.

SEM – standard error of the mean.

In terms of baseline laboratory values, total white count, HbA1C levels, C-reactive protein levels, and cholesterol profiles were similar at baseline between the groups. However, at follow-up the total white count in the statin arm was higher than controls (8.8+/−1.4 vs 6.3+/−1.9 10^9^ cells/L, p<0.01, **Table 2**). Furthermore, as expected both total and LDL cholesterol levels were lower in the statin arm (2.9+/−1.0 vs. 4.6+/−1.0 and 1.3+/−0.7 vs. 3.0+/−1.0 mmol/L respectively, p<0.01).

**Table 2:** Laboratory characteristics of patients.

	Control Group	Statin Group	Healthy	p-value
	(n = 10)	(n = 10)	(n = 15)	
*Baseline*				
WBC (SEM)	7.2 (1.5)	8.0(2.4)	6.9 (1.5)	NS
HbA1C	0.052 (0.005)	0.051 (0.003)	0.053 (0.003)	NS
C-reactive protein	6.8 (11.4)	8.3 (9.0)	**1.9 (1.1)**	p<0.05
Total cholesterol	5.3 (0.7)	4.8 (1.0)	5.0(1.0)	NS
HDL	1.0 (0.3)	1.0 (0.1)	1.2 (0.3)	NS
LDL	3.3 (0.6)	2.9 (0.9)	3.3 (1.0)	NS
TG	2.1 (1.0)	1.9 (1.2)	**1.3 (0.9)**	p<0.05
*Follow-up*				
WBC	6.3 (1.9)	**8.8 (1.4)**		p<0.01
HbA1C	0.053 (0.001)	0.054 (0.001)		NS
C-reactive protein	6.5 (8.2)	21.7 (6.9)		NS
Total cholesterol	4.6 (1.0)	**2.9 (1.0)**		p<0.01
HDL	1.0 (0.2)	0.9 (0.2)		NS
LDL	3.0 (1.0)	**1.3 (0.7)**		p<0.01
TG	1.6 (0.5)	1.5 (0.7)		NS

WBC – white blood cell 10^9^ cells/L.

HbA1C – hemoglobin A1C.

HDL – high density lipoprotein mmol/L.

LDL – low density lipoprotein mmol/L.

TG – triglycerides mmol/L.

SEM – standard error of the mean.

### Effect of statin therapy on circulating EPC and CAC levels

#### a) Baseline Flow Characteristics

**Figure 1:**
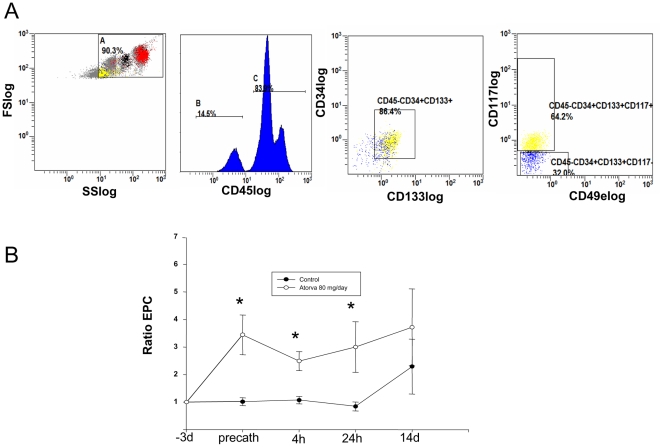
Flow cytometric analysis of CD45dimCD34+CD133+CD117+ endothelial progenitor cells (EPC). (**A**) Sample gating strategy. (**B**) EPC levels, expressed at each interval as a ratio to baseline levels (taken three days prior to PCI) for patients receiving and not receiving Atorvastatin therapy (n = 10 per group). * indicates p<0.05.

CD45dim EPC were enumerated at baseline in a total of 47 individuals, 32 with CAD and 15 healthy controls (**Figure 1A**). While healthy controls had nearly three fold more EPC than CAD patients the difference did not reach significance (12.7+/−2.7 vs 5.4+/−0.6 cells/µL, p = 0.07). Patients who had been referred for angiography after presenting with an acute coronary syndrome (ACS) in the previous 14 days did not have more EPC at baseline than non-ACS patients (5.0+/−1.0 vs. 5.6+/−0.6 cells/µL, p>0.05).

#### b) Serial Flow Cytometric Assessment of Cell Profiles

When looking at the effects of PCI on mobilization of EPC we compared the abundance of this cell type at the following intervals: three days prior to catheterization, pre-catheterization, 4 hrs, 24 hrs and 14 days. While EPC levels did not fluctuate over time for the patients who did not receive Atorvastatin, there was a 3.5-fold increase in EPC levels with high dose Atorvastatin within 3 days of the first dose (immediately pre-PCI) that persisted at 4 and 24 hours post-PCI (p<0.05). There was no effect seen on EPC levels caused by PCI (**Figure 1B**). Additionally, we serially assessed CD49e expression on circulating EPCs and found low levels of basal expression and both the number of CD49e+ cells nor the relative CD49e expression was effected by statin therapy or PCI (data not shown).

#### c) *In vitro* CAC Assessment

**Figure 2:**
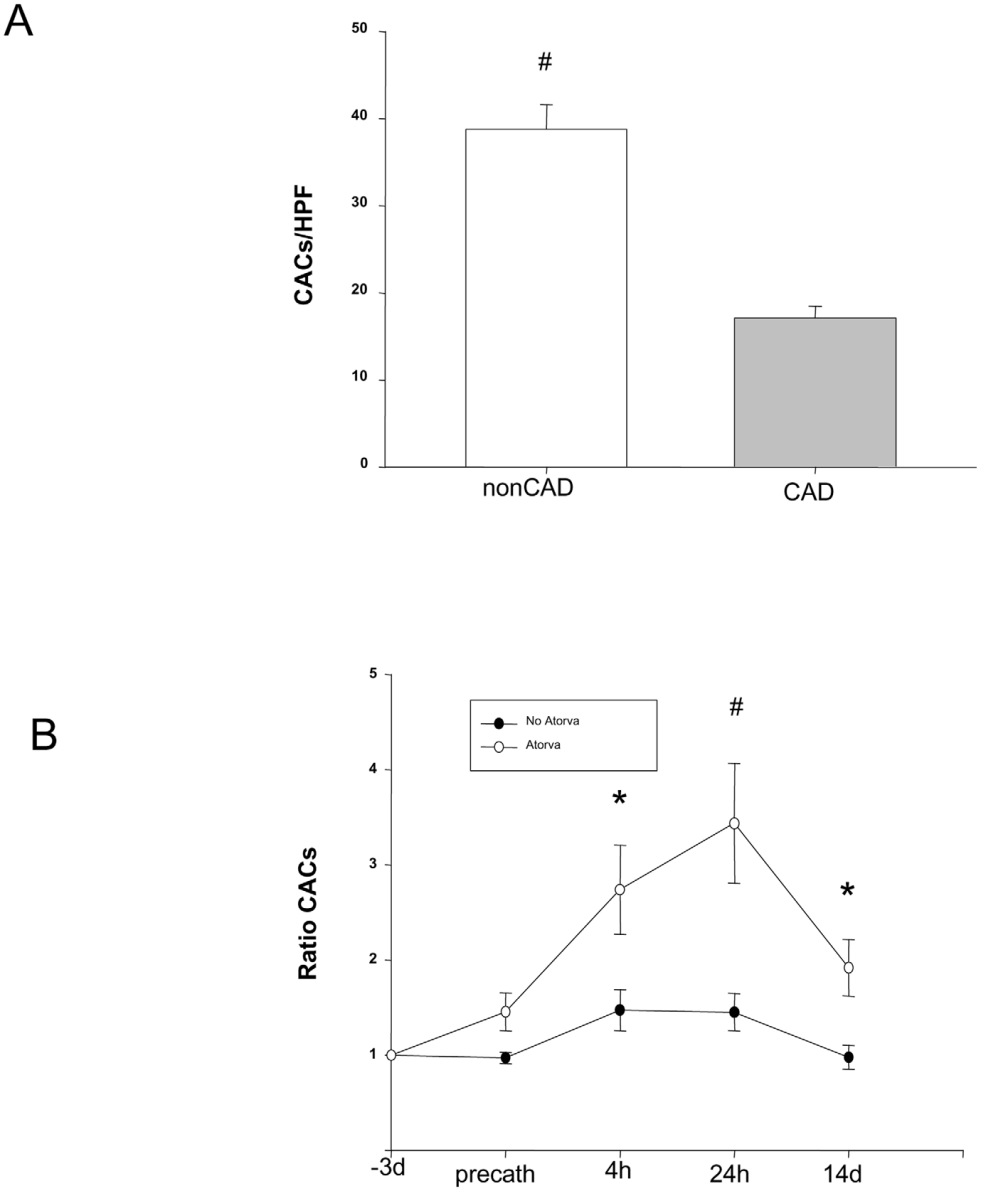
*In vitro* assessment of cultured angiogenic cells (CAC). (**A**) Comparison of baseline CAC levels in patients with CAD (n = 32) *vs*. healthy controls (non-CAD). (**B**) CAC levels expressed at each interval as a ratio to baseline levels (taken three days prior to the procedure) for patients receiving and not receiving Atorvastatin therapy (n = 10 per group). * indicates p<0.05, # indicates p<0.01.

CAC were enumerated by culture assay and yielded similar results to those obtained by flow cytometry. Baseline CAC levels were 2-fold higher in healthy controls compared to patients with CAD at baseline (e.g., 38.8+/−2.3 *vs*. 17.1+/−1.5, p<0.01, **Figure 2A**). Again, there were no differences in CAC levels between patients with a recent ACS compared to those with clinically stable symptoms (p<0.05, data not shown). Interestingly, a significant interaction was seen between Atorvastatin therapy and PCI when precatheterization, 4 hour and 24 hour levels were assessed (2 way ANOVA: p = 0.006 for Atorvastatin and Time from PCI interaction). However, in those patients receiving Atorvastatin, there was an early increase in CAC levels until 24 hours post-PCI followed by a tapering off effect – although 14 day levels remained approximately 2-fold higher than baseline (e.g., 4 h: 2.7+/−1.5, 24 h: 3.4+/−0.6, 14 d: 1.9+/−0.3, **Figure 2B**).

### Effect of statin therapy on function of CAC

**Figure 3:**
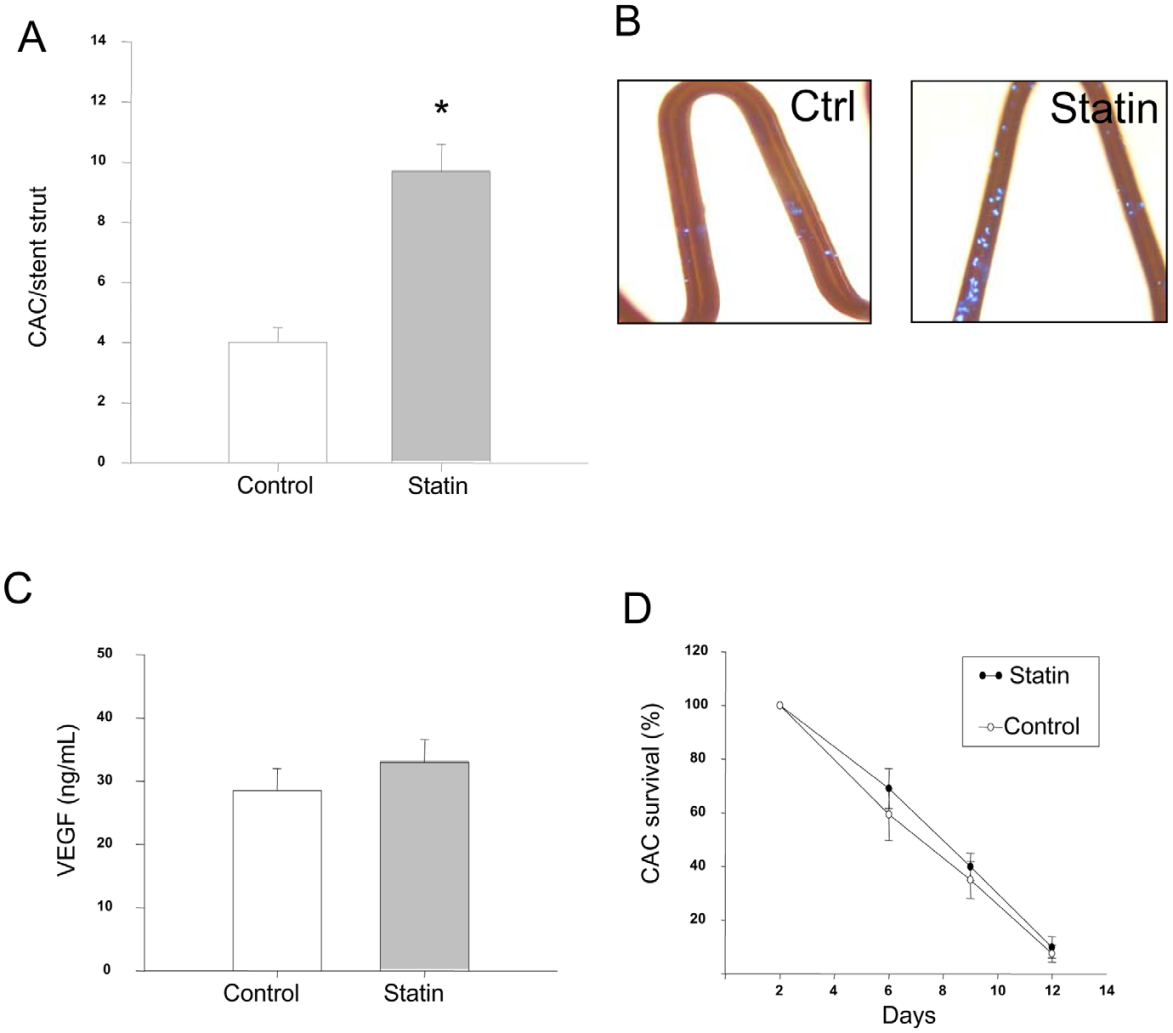
Effect of statin on cultured angiogenic cell (CAC) function *in vitro*. (**A**) Atorvastatin supplementation of the media (0.1 mM). Statin treatment improves CAC attachment to bare metal stent struts *in vitro* (n = 10). * indicates p<0.01. (**B**) Representative photos showing DAPI nuclear staining of CAC attachment to stent struts. (**C**) Atorvastatin supplementation of the media at 0.1 mM does not effect VEGF secretion by EPCs (n = 6, p>0.05). (**D**) Statin does not ameliorate survival of CACs in culture (n = 6, p>0.05).

Statin supplementation of the culture media at a concentration of 0.1 µM failed to alter survival of CAC *in vitro* (**Figure 3D**, p>0.05). Similarly, secretion of the angiogenic cytokine VEGF was unaffected by culture in the presence of Atorvastatin in the culture media (e.g., 28.5+/−3.5 vs 32.9+/−3.6 ng/mL, **Figure 3C**, p>0.05). Finally, CAC adhesion to BMSs was tested *in vitro* by co-culture of CAC in the presence of a bare metal coronary stent immobilized in collagen. After 48 hours *in vitro*, co-culture with Atorvastatin augmented CAC attachment to stents by 2-fold compared to cells co-cultured in the absence of Atorvastatin (e.g., 9.7+/−0.9 vs. 4.0+/−0.6, n = 10, p<0.01, **Figure 3A,B**). As integrins are known to be important modulators of CAC adhesion[19], [26] we assayed by RT-qPCR the expression of the α1-5 subunits of the integrin family and found no significant regulation of mRNA levels (p>0.05, data not shown).

## Discussion

While it is empirically recognized that statins may increase EPC levels in patients, the temporal course and profile of the cells mobilized by statins at time of PCI had not previously been characterized. Nonetheless, patients undergoing the implantation of stents designed to trap CD34+ cells (e.g., Genous endothelial progenitor cell capturing stent) are routinely pre-treated with high dose statin therapy with the hope of augmented circulating EPC levels.[27] This study is the first attempt to prospectively randomize patients undergoing stent insertion to high dose statin therapy and describe in a serial fashion the time course and extent of EPC and CAC mobilization. Our data demonstrates that 80 milligrams of Atorvastatin per day, beginning three days prior to PCI, is associated with a mobilization of both EPC and CAC. As well, this is the first study to enumerate the highly selective population of CD34+/CD117+ (c-kit) progenitors by flow cytometry in patients undergoing revascularization. Recently c-kit has been found to define a population of human coronary vascular progenitor cells that are capable of regenerating competent coronary vessels and improving coronary blood flow.[15] Finally, we demonstrate that statin treatment improves the functional capacity of CAC, by augmenting attachment to stent struts using a novel *in vitro* model. It is important to note that this increase in EPC adhesion was not due modulation to member of the integrin family and similar to *in vivo* observations by Banerjee *et al*.,[28] occurred without a change in VEGF levels.

Results from the studies of EPCs and PCI continue to yield equivocal or discordant results.[29] This likely reflects both the varying manner in which EPCs are quantified as well as limitations in study design. For example, the largest study to date was performed by Inoue *et al.* who looked at 40 patients undergoing PCI.[30] They studied both CD34+ cells by flow cytometry and outgrowth of either endothelial or smooth muscle cells (SMCs) from PBMCs at multiple time points out to one month post PCI. They noticed an association between mobilization of CD34+ cells and outgrowth of SMCs with ISR but by limiting their flow cytometry to a single marker they most certainly included all early bone marrow derived cells including hematopoietic progenitors cells. Mills et al. examinined circulating EPC number and colony forming unit mobilization in 24 patients undergoing elective PCI.[31] Similar to our data, they observed a 3-fold increase in cultured cells in the first 24 hours following PCI with the majority of their patients being concomitantly treated with statin medications. Finally, Egan *et al.* [32] compared 10 patients undergoing PCI with 13 patients having angiogram only and found that CXCR4 positive cells were mobilized in response to PCI when compared to angiography alone. In contrast, our study looked at multiple time points out to two weeks after PCI in a total of 20 patients between both arms. More importantly, our data is the first to randomize patients to statin medications and to serially assess their EPC levels – providing a novel mechanistic link between the observed clinical outcomes and early statin therapy in patients undergoing PCI.

As in the literature regarding PCI and EPCs, the effect of statin therapy on both initial mobilization and maintenance of EPC levels has yielded sometimes conflicting results. Vasa and colleagues first reported an increase in EPCs (CD34+/KDR+) and CACs out to 28 days in 40 patients with CAD.[33] Most recently, these results were replicated in a small cohort of patients initiated on Atorvastatin therapy, yielding a doubling of CD45dim/CD34+/KDR+ cells at four weeks.[34] While neither of these studies were randomized or had control groups for comparison, their results are very similar to those observed in the current study. Paradoxically, in patients with CAD Hristov *et al.* [35] quantified both EPCs and CACs in a large non-randomized cohort and noted a decrease in EPCs during chronic statin therapy (especially at higher doses). Similarly, Deschaseaux et al. [36] derived fewer colony forming units from patients on chronic statin therapy than a matched cohort, suggesting the initial mobilization observed may not be sustained over longer periods. However, the non-randomized nature of these studies and the lack of standard dosing certainly justifies trepidation in drawing firm conclusions. Data from large randomized studies of both early and longterm effects of statin therapy on EPCs is clearly warranted.

Perhaps the most intriguing finding of our study was the early difference in EPC levels seen with Atorvastatin loading – an effect that was observed with both flow cytometry and CAC culture which persisted for at least 24 hours post-PCI. However, by 14 days EPC levels had tapered off somewhat – yet remained elevated relative to baseline levels. The cause of the diminution of EPCs by day 14 is unclear, but perhaps suggests that the mobilization of EPCs by statins is a time-limited, transient effect. Alternatively, there may be either no further EPCs available to be mobilized or some other rate-limiting process is preventing EPCs from being generated and/or mobilized by statins. Interestingly, there was a noticeable rise in EPC levels in the control arm at 14 days. This likely in part reflects more aggressive management of patients atherosclerotic disease and risk factors in patients newly identified with CAD. For example, smoking cessation has been associated with and early and robust rise in EPC levels.[37] Similarly complimentary pharmacotherapy such as angiotensin converting enzymes[38] or thiazolidinediones (glitazones)[39] are also known to increase EPC levels. Thus, this late rise in the control patients most likely represents improved risk factor modification.

It is important to realize that our protocol is similar to those used in the ARMYDA,[40] ARMYDA-ACS,[41] and the more recently completed ARMYDA-RECAPTURE[42] studies. Both ARMYDA and ARMYDA-ACS demonstrated a clear reduction in peri-procedural myocardial infarctions in statin naïve patients undergoing PCI for stable coronary artery disease or acute coronary syndromes respectively when high dose Atorvastatin was initiated prior to the procedure. Perhaps more clinically relevant is the ARMYDA-RECAPTURE study in which the investigators reloaded patients already on statins with 80 mg of Atorvastatin and showed reductions in peri-procedural myocardial infarctions. Finally, in the recently published PCI-PROVE IT study that compared the frequency of major adverse cardiac events in patients undergoing PCI and treated with either moderate (Pravastatin 40 mg/day) or intensive (Atorvastatin 80 mg/day) statin therapy there was a clear cut advantage for the patients treated in the intensive therapy arm.[11] For example, the primary composite end point (death from any cause, myocardial infarction, documented unstable angina requiring hospitalization and revascularization at least 30 days after randomization, and stroke) showed a 22% relative risk reduction with intensive statin therapy (p = 0.001) Moreover, the intensive statin therapy reduced target vessel revascularization (p<0.001) – an effect that persisted (p = 0.015) after adjusting for 30-day on treatment serum LDL-C and CRP concentrations. Clearly the rapid clinical benefits seen in these studies involving intensive statin therapy suggests a mechanism of action that is independent of LDL-lowering. Given the similarities of these studies to our current protocol, it is attractive to hypothesize that the benefit of acute Atorvastatin loading prior to PCI may in part be derived from the off-target effects of statins on EPC mobilization and function.

Certainly, our study is not without limitations. For example, given the widespread use of statins in patients with risk factors for established CAD, it proved difficult to recruit statin naïve patients for this study. Therefore, while our sample size allows us to confidentially address questions specific to serial changes in EPCs, it lacks sufficient power to address the relationship between EPC mobilization profiles and clinical outcomes (e.g., ISR or peri-procedural infarctions). Furthermore, given the relatively small patient population, it is unclear if this observation is applicable in populations in whom statin therapy has failed to show a benefit – such as patients on hemodialysis.[43], [44]

In summary, our data demonstrate early benefit of statin therapy on EPC number and CAC number and function in humans undergoing PCI. Studies aimed to understand mechanisms by which EPCs are mobilized during PCI and the potential benefits that these cells confer will likely provide novel targets and therapies for improving clinical outcomes in patients undergoing revascularization.
